# Influence of language barrier and cultural background in hepatitis B disease knowledge in a Chinese community of Spain

**DOI:** 10.3389/fpubh.2024.1324336

**Published:** 2024-04-10

**Authors:** Anna Pocurull, Cristina Collazos, Anna Miralpeix, Laura Tapias, Tao Wang, María Jose Moreta, Zoe Mariño, Sabela Lens, Xavier Forns

**Affiliations:** ^1^Liver Unit, Hospital Clínic de Barcelona, IDIBAPS, University of Barcelona, Barcelona, Spain; ^2^Centro de Investigación Biomédica en Red de Enfermedades Hepáticas y Digestivas (CIBERehd), Madrid, Spain

**Keywords:** chronic hepatitis B, language barrier, hepatitis B knowledge, cultural background, hepatitis B vaccination

## Abstract

**Introduction:**

Hepatitis B infection (HBV) is prevalent in China. Due to language barriers and cultural differences, it is not always straightforward to evaluate disease knowledge in liver clinics. We aimed to assess the awareness on HBV and its mechanisms of transmission in HBV-infected Chinese patients and their household contacts.

**Methods:**

HBV-infected Chinese patients and their contacts were interviewed by a native Chinese nurse regarding their knowledge on HBV transmission mechanisms, use of preventive measures and vaccination status. Non-Chinese HBV-infected patients and their household contacts served as a control group.

**Results:**

In total 182 patients and 398 contacts participated with 85 (47%) patients and 240 (60%) contacts being from China. Language barrier was reported in 80% of Chinese patients and 44% of their contacts. Knowledge on parenteral and sexual HBV transmission was high in all patients (~90%) but Chinese were more aware of vertical transmission than controls (94% vs. 68%; *p* < 0.01). Regarding the use of preventive measures, Chinese patients were more forewarned in their use to avoid parenteral transmission (93% vs. 74%, *p* < 0.01). When assessing household contacts, Chinese used preventive measures more frequently than controls for parenteral and sexual transmission (79% vs. 65 and 81% vs. 48%, *p* < 0.05). Vaccination coverage was slightly lower in Chinese contacts compared to controls (78% vs. 86%, *p* = 0.05).

**Conclusion:**

Despite relevant language barriers, Chinese patients are well informed on the mechanisms of HBV transmission. Cultural differences may explain a higher use of preventive measures among the Chinese population. HBV vaccination of household contacts should be reinforced in both groups.

## 1 Introduction

Hepatitis B is a potentially life-threatening liver infection and a major global health problem (1, 2). In highly endemic areas, hepatitis B virus (HBV) is most commonly spread by perinatal transmission or through parenteral transmission (exposure to infected blood) (3, 4), while in less endemic areas is commonly spread sexually (5–8). Familiar transmission has also been described (9, 10), most likely due to the sharing of contaminated materials (11). The widespread use of hepatitis B vaccine in infants has considerably reduced the incidence of new chronic infections.

Due to the high prevalence of HBV infection in individuals from Asian origin (12), they represent a significant proportion of patients followed in Liver Disease Units in Spain. The Chinese community in Barcelona has grown considerably in the last 20 years and the last official data (2021) (13) indicate that more than 22.000 Chinese people are censored in the city. Importantly, around one third live in the area of influence of our University Hospital. Access to Health-Care services is not optimal in this population; indeed it is quite frequent that the first contact of Chinese individuals with the Health System is during pregnancy (when HBsAg is screened) (14). Given the relevant language barriers and cultural differences, we hypothesized that Chinese HBV-infected patients and their relatives might be less aware on the mechanisms of HBV transmission and vaccine coverage.

Our aim was to measure the awareness on hepatitis B mechanisms of transmission in HBV-infected Chinese patients and household contacts, and to assess their vaccination status.

## 2 Methods

This is an observational case–control study. All patients with a diagnosis of chronic hepatitis B of Chinese origin followed at our Liver Unit from 2020 to 2022 were invited to participate. A similar number of non-Chinese HBV-infected patients were used as a control group. The only exclusion criteria was refusal to participate in the study. The following variables were recorded for each patient: age, sex, initial HBV diagnosis, current antiviral therapy, HBV-DNA levels, last transient elastography value and the presence of cirrhosis.

All patients and their household contacts were contacted telephonically by personnel of the study (LT, TW) to undergo an interview. To avoid potential language and cultural barriers a Chinese nurse (TW) contacted Chinese patients and their contacts. In case a patient/household contact did not speak Mandarin, family members helped in the translation. For patients, the interview included a question on their level of Spanish, level of education, knowledge on the mechanisms of HBV transmission (parenteral, sexual, vertical) and use of preventive measures for HBV transmission (Supplementary Table S1). For household contacts the interview included demographic data, familiar relationship with the patient, level of Spanish, knowledge on the mechanisms of HBV transmission (parenteral, sexual, vertical), the use of preventive measures and the status of HBV vaccination (Supplementary Table S1). The question regarding the use of preventive measures for sexual transmission was only asked to patients’ partners. Children below age 14 were not interviewed, and only their vaccination status was recorded.

### 2.1 Statistical analysis

Relative frequency is expressed in percentage. Quantitative variables are depicted as median (percentiles 25 and 75).

Regarding qualitative variables, differences between groups were calculated by the Chi-square test. For quantitative variables, differences between groups were calculated by Student’s t-test. Multivariate analysis was conducted using logistic regression, including variables exhibiting a *p* value <0.1 in the univariate analysis. Individuals who did not provide an answer were not included in the analysis. A *p*-value <0.05 was considered statistically significant. All computations were carried out in IBM SPSS Statistics version 21.0 (IBM, Chicago, USA).

### 2.2 Ethics

The study was approved by Hospital Clínic Barcelona Ethics Committee (HCB/2021/0752) and informed consent was obtained from all the participants and from the legal guardians of the participants who were below 18 years of age. The consent was written in Chinese for patients of this origin. An oral authorization from all patients was obtained to allow us to contact their relatives, who also gave oral approval to be interviewed. All data was registered anonymously.

## 3 Results

### 3.1 Characteristics of patients and household contacts

From 142 HBV-infected Chinese patients with records in our database, 2 did not give their consent to participate and 55 were not reached after 3 phone call attempts in different days. Thus, 85 Chinese patients were finally included in the study.

In the control group, from the initial 142 consecutive HBV-infected Caucasian patients 3 declined to participate, 6 were dead and 36 were not reached after 3 phone call attempts. Thus, 97 non-Chinese patients were finally included in the study.

The main characteristics of the patient cohorts are depicted in Table 1. Age did not differ between the two groups, but female sex was significantly higher in the Chinese cohort. In both groups, the most common diagnosis was HBeAg-negative Chronic Hepatitis B infection (around 50%). The proportion of patients under NAs (nucleos(t)ide analogs) therapy was significantly greater for Chinese (48%) compared to control (36%). HBV-DNA was detectable in 50% of patients, similar between groups.

**Table 1:** Baseline characteristics of patients.

Patients	All patients *N* = 182	Chinese *N* = 85	Non Chinese *N* = 97	*p*
Gender (F)	77 (42%)	43 (51%)	34 (35%)	0.03
Age (Y)	46 (39–52)	42 (36–52)	48 (43–53)	0.05
Initial diagnosis				<0.01
HBeAg negative infection	90 (50%)	37 (43%)	53 (55%)	
HBeAg positive infection	9 (5%)	9 (11%)	0 (0%)	
HBeAg positive chronic hepatitis	32 (17%)	25 (29%)	7 (7%)	
HBeAg negative chronic hepatitis	29 (16%)	14 (17%)	15 (16%)	
HBV acute hepatitis	13 (7%)	0	13 (13%)	
HBV/HDV coinfection	9 (5%)	0	9 (9%)	
HBV-DNA (detectable)	90 (49%)	46 (54%)	44 (45%)	0.27
HBV-DNA (IU/mL) **	396 (17–4,780)	1,090 (12–25,800)	228 (0–2,545)	0.03
Antiviral Treatment (yes)	76 (42%)	41 (48%)	35 (36%)	0.02
Transient elastography (kPa)	5.8 (4–6)	5.6 (4–6)	6 (4–6)	0.37
Cirrhosis (yes)	27 (15%)	8 (9%)	19 (20%)	0.05

Quantitative: median (IQR), qualitative: number (%). ** Patients not receiving NAs.

A total of 398 household contacts were telephonically interviewed, 60% Chinese and 40% Caucasian.

### 3.2 Knowledge on HBV transmission and use of preventive measures in HBV-infected patients

Among Chinese patients 68 of 85 (80%) acknowledged major limitations in the understanding of Spanish. The level of education was significantly higher in the control group than in Chinese patients (Table 2).

**Table 2:** Knowledge and attitude regarding HBV transmission and comparison between Chinese vs. non-Chinese patients.

Patients	All patients *N* = 182	Chinese *N* = 85	Non Chinese *N* = 97	*p*
Spanish understanding				< 0.01
No	26 (14%)	25 (29%)	1 (1%)	
Limited	47 (26%)	43 (51%)	4 (4%)	
Good	109 (60%)	17 (20%)	92 (95%)	
Level of education (*n* = 126)				0.01
Primary education	45 (36%)	30 (57%)	15 (21%)	
High School	47 (37%)	19 (36%)	28 (38%)	
University degree	34 (27%)	4 (7%)	30 (41%)	
Knowledge on parenteral transmission				0.05
No	12 (6%)	9 (10%)	3 (3%)	
Yes	170 (94%)	76 (90%)	94 (97%)	
Knowledge on sexual transmission				0.05
No	14 (8%)	10 (12%)	4 (4%)	
Yes	168 (92%)	75 (88%)	93 (96%)	
Knowledge on vertical transmission				< 0.01
No	36 (20%)	5 (6%)	31 (32%)	
Yes	146 (80%)	80 (94%)	66 (68%)	
Prevention measures (parenteral transmission) *n* = 179				< 0.01
No	31 (17%)	6 (7%)	25 (26%)	
Yes	148 (83%)	76 (93%)	72 (74%)	
Prevention measures (sexual transmission) *n* = 181				0.14
No	73 (40%)	29 (34%)	44 (45%)	
Yes	108 (60%)	55 (66%)	53 (55%)	
Reason for sexual prevention *n* = 86				0.38
Avoid pregnancy	39 (45%)	22 (45%)	17 (46%)	
Sexual prevention	47 (55%)	27 (55%)	20 (54%)	

Overall awareness on parenteral and sexual HBV transmission was good (~90%), with better results in the control group compared to Chinese patients, but not reaching statistical significance (*p* = 0.05 for both variables). Males were more aware on sexual transmission than females (97% vs. 86%) (Table 3) and male sex was the only variable independently associated with a better knowledge on HBV sexual transmission (OR 5.02 CI 95% 1.3–18.9; *p* = 0.02) (Supplementary Table S2).

**Table 3:** Knowledge of transmission routes and preventive measures according to clinical, demographic, and virological characteristics in patients.

Knowledge on mechanisms of HBV transmission										Preventive measures for HBV transmission					
Variables *N* = 182	Parenteral transmission		*p*	Sexual transmission		*p*	Vertical transmission		*p*	Parenteral transmission		*p*	Sexual transmission		*p*
	Yes	No		Yes	No		Yes	No		Yes	No		Yes	No	
Age (Y)	45 (39–52)	49 (36–56)	0.37	46 (39–52)	46 (39–60)	0.33	45 (38–52)	47 (45–64)	0.02	46 (39–53)	45 (33–51)	0.20	45 (38–52)	47 (40–54)	0.02
Female (*n* = 77)	70/77 (91%)	7/77 (9%)	0.25	66/77 (86%)	11/77 (14%)	0.01	69/77 (90%)	8/77 (10%)	0.01	61/76 (80%)	15/76 (20%)	0.46	48/76 (63%)	28/76 (37%)	0.42
Male (*n* = 105)	100/105 (95%)	5/105 (5%)		102/105 (97%)	3/105 (3%)		77/105 (73%)	28/105 (27%)		87/103 (85%)	16/103 (15%)		60/105 (57%)	45/105 (43%)	
Chinese (*n* = 85)	76/85 (90%)	9/85 (10%)	0.05	75/85 (88%)	10/85 (12%)	0.05	80/85 (94%)	5/85 (6%)	0.01	76/82 (93%)	6/82 (7%)	0.01	55/84 (66%)	29/84 (34%)	0.14
Non-Chinese (*n* = 97)	94/97 (97%)	3/97 (3%)		93/97 (96%)	4/97 (4%)		66/97 (68%)	31/97 (32%)		72/97 (74%)	25/97 (26%)		53/97 (55%)	44/97 (45%)	
Primary studies (*n* = 45)	38/45 (84%)	7/45 (16%)	0.09	39/45 (87%)	6/45 (13%)	0.28	39/45 (87%)	6/45 (13%)	0.07	36/44 (82%)	8/44 (18%)	0.39	22/45 (49%)	23/45 (51%)	0.32
Advance studies* (*n* = 81)	76/81 (94%)	5/81 (6%)		75/81 (93%)	6/81 (7%)		59/81 (73%)	22/81 (27%)		60/80 (75%)	20/80 (25%)		47/81 (58%)	34/81 (42%)	
Treatment (*n* = 76)	68/76 (89%)	8/76 (11%)	0.07	68/76 (89%)	8/76 (11%)	0.22	NA	NA		65/74 (85%)	9/74 (15%)	0.13	48/76 (63%)	28/76 (37%)	0.42
No treatment (*n* = 106)	102/106 (96%)	4/106 (4%)		100/106 (94%)	6/106 (6%)					83/105 (79%)	22/105 (21%)		60/105 (57%)	45/105 (43%)	
Positive DNA-HBV (*n* = 90)	86/90 (95%)	4/90 (5%)	0.25	84/90 (93%)	6/90 (7%)	0.61	NA	NA		70/89 (79%)	19/89 (21%)	0.16	52/89 (58%)	37/89 (42%)	0.74
Negative DNA-HBV (*n* = 92)	84/92 (91%)	8/92 (9%)		84/92 (91%)	8/92 (9%)					78/90 (87%)	12/90 (13%)		56/92 (61%)	36/92 (39%)	

Denominators refer to the number of patients that provided information. Around 30% of patients did not answer about their educational level. *Advance studies: High school or university; NA, Non- applicable.

Awareness on HBV vertical transmission was significantly better in Chinese patients (94%) compared to controls (68%, *p* < 0.01). Younger age and female sex were also associated with a better knowledge on vertical transmission (Table 3), but the only variable independently related to knowledge on vertical transmission by multivariate analysis was Chinese origin (OR 4.9 CI 95% 1.5–16.2, *p* = 0.01) (Supplementary Table S2).

Regarding the use of preventive measures to avoid parenteral HBV transmission (sharing scissors, toothbrush, or manipulation of infected patients’ wounds) being Chinese was the only variable associated with its use (93% for Chinese vs. 74% for controls, *p* < 0.01); we did not find any relationship with age, sex, or being on NAs therapy. Concerning preventive measures to avoid sexual HBV transmission, only a younger age was associated with a significantly higher use of preventive measures (Table 3). It is important to notice, however, that prevention of pregnancy was the reason for using barrier methods in ~50% of patients (Table 2).

### 3.3 Knowledge on HBV transmission and use of preventive measures in household contacts

For household contacts, the proportion of individuals with major limitations in the understanding of Spanish decreased to 44%.

As expected, most (96%) household contacts who answered the survey knew about HBV infection of the index patient. Among them, awareness on parenteral and sexual transmission was excellent among control individuals (96 and 95%, respectively) and fair among Chinese (86 and 81%, respectively); these differences reached statistical significance (Supplementary Table S3). Both groups had a similar awareness on HBV vertical transmission (>80%). Younger age was found to be associated with increased awareness of vertical transmission (Supplementary Table S4).

As we found in patients, the use of preventive measures to avoid parenteral HBV transmission was significantly higher in Chinese household contacts (Supplementary Tables S3, S4), being the only variable associated with its use. Concerning preventive measures to avoid sexual HBV transmission, we only interviewed patients’ partners. We found a significantly higher use of preventive methods in Chinese (81%) than in control partners (48%, *p* < 0.01) (Supplementary Table S3).

Antiviral treatment (patients) or vaccination status (household contacts) did not influence the use of preventive measures (Supplementary Table S5).

### 3.4 Vaccination status among household contacts

When asked about their vaccination status, 74 (19%) household contacts reported not being fully vaccinated (three completed doses) (Supplementary Table S3). When comparing both groups, household contacts of Chinese patients had a slightly lower vaccination rate compared to controls (78% vs. 86%, *p* = 0.05). Importantly, when household contacts of Chinese patients were categorized by place of birth, the rate of vaccination was 96% if born in Spain compared to only 59% if born in China (*p* < 0.01).

## 4 Discussion

HBV is a highly transmissible virus and thus, knowledge on the potential mechanisms of transmission and measures to prevent it are relevant. Our initial hypothesis was that in migrants from areas of high HBV prevalence, language barriers and cultural differences might difficult engagement into local healthcare and educational programs and consequently, decrease awareness on diagnosis and prevention of hepatitis B (15, 16).

Contrarily to our hypothesis, we found that Chinese patients were aware of the routes of HBV transmission. Differences between Chinese and control patients were slight regarding the parenteral and sexual routes, but patients from China had a significantly better knowledge on the potential vertical transmission of the virus. The different epidemiology of HBV infection in Asia (mainly transmitted from mother-to-child) compared to Europe (where HBV is mainly transmitted by the sexual route) is the most likely explanation, since origin (and not educational level or sex) was the only variable independently associated with vertical transmission awareness. Our data seem to be in accordance with data published by Huang et al. (17) and suggest that information on HBV transmission mechanisms in Chinese patients were acquired in China.

When the same questions were asked to household contacts, we found that awareness of parenteral and sexual transmission mirrored the pattern seen in patients, with a slightly (but significant) lower knowledge among Chinese individuals. Interestingly, the better knowledge on HBV vertical transmission of Chinese patients was lost for household contacts. Since half of them were born in Spain, the latter may just reflect the epidemiology of HBV infection in our country, with a prevalence below 1%, and practical absence of vertical transmission (18).

Regarding the use of preventive measures to avoid HBV transmission, we found that Chinese patients and their relatives were more prone to use them. This finding is against our initial hypothesis, since we believed that language barriers would negatively impact the use of preventive measures. One potential explanation might be that Asian population is well instructed in the use of barriers to avoid infections (not only those transmitted by the parenteral route but also by air). Moreover, stigma caused by HBV infection is still a problem in mainland China (19), where the infection is associated with fear of contagion (20). Thus, we believe that our findings mainly reflect different cultural backgrounds between Asia and Europe.

Vaccine coverage was overall good; however, when comparing HBV vaccination in relatives from Chinese and native patients, we found a lower (although not significant) vaccination rate in the first group. Nevertheless, when better dissecting the vaccination rates among household contacts of Chinese patients, we found a practical universal coverage in relatives born in Spain (where HBV vaccination is universal since 1991), compared to rates below 60% in those born in China. The latter reflects the lower vaccination coverage in China in the adult population (21). Thus, results from our study emphasize the need to (22) further promote vaccination among this group of individuals at risk.

The study has some limitations. First, a sample size was not estimated due to the lack of solid data in the literature to establish a hypothesis on the expected differences between Chinese and Non-Chinese patients. Second, around 30% of household contacts were not available for the interview, and we cannot exclude that this introduced a selection bias. Second, all collected data are self-reported and interviews were performed by phone, which might not be as accurate as face-to-face (21). Another limitation is that lack of validation of our questionnaire; however, we based our interview on well-known mechanisms of HBV transmission and followed the main items described by the Centers for Disease Control and Prevention (21). Finally, questions about potential misconception on HBV transmission, such as transmission by shaking hands or sharing food were not included in the questionnaire (17).

## 5 Conclusion

In summary, we found that, despite the presence of relevant language barriers, Chinese HBV-infected patients are well informed on the mechanisms of HBV transmission. Chinese patients and household contacts used preventive measures more frequently, which might be explained by cultural differences. An important finding was the low rate of HBV vaccination among household contacts born in China, which requires a targeted public health intervention.

## Data availability statement

The raw data supporting the conclusions of this article will be made available by the authors, without undue reservation.

## Ethics statement

The studies involving humans were approved by Ethics commitee Hospital Clínic Barcelona. The studies were conducted in accordance with the local legislation and institutional requirements. Written informed consent for participation in this study was provided by the participants’ legal guardians/next of kin. Written informed consent was obtained from the minor(s)’ legal guardian/next of kin for the publication of any potentially identifiable images or data included in this article.

## Author contributions

AP: Conceptualization, Data curation, Formal analysis, Methodology, Validation, Visualization, Writing – original draft. CC: Data curation, Validation, Visualization, Writing – review & editing. AM: Data curation, Validation, Visualization, Writing – review & editing. LT: Validation, Visualization, Data curation, Writing – review & editing. TW: Data curation, Validation, Visualization, Writing – review & editing. MM: Data curation, Validation, Visualization, Writing – review & editing. ZM: Data curation, Investigation, Methodology, Validation, Visualization, Writing – review & editing. SL: Data curation, Investigation, Methodology, Supervision, Validation, Visualization, Writing – review & editing. XF: Data curation, Formal analysis, Funding acquisition, Project administration, Resources, Supervision, Validation, Visualization, Writing – original draft, Writing – review & editing.
