# Supplementary material for: Influence of language barrier and cultural background in hepatitis B disease knowledge in a Chinese community of Spain
Source: Front Public Health. 2024 Apr 10;12:1324336. doi: 10.3389/fpubh.2024.1324336 (PMC11040686; doi:10.3389/fpubh.2024.1324336)
Supplement: Supplementary file 1 [file Data_Sheet_1.PDF]

Supplementary Table 1 . Patients' and contacts' interview

(a) Language barrier

a.1 What is your level of Spanish understanding? (No, Limited, Yes)

a.2 What is your level of Catalan understanding? (No, Limited, Yes)

(b) Knowledge about transmission mechanisms

b.1 Do you know the possibility of hepatitis B virus parenteral transmission through infected blood (sharing scissors, tooth brush, or manipulation of infected patients' wounds)? (Yes, No)

b.2 Do you know the possibility of hepatitis B virus transmission by sexual relationships? (Yes, No)

b.3 Do you know that hepatitis B virus can be transmitted from mother to child during pregnancy/delivery?(Yes, No)

(c) Have you informed your relatives/household contacts about your hepatitis B? (Informed, Not informed)

(d) Use of preventive measures

d.1 Do you use of preventive measures to avoid exposure to infected blood (such as avoiding sharing personal items) (Yes, No)

d.2 Do you use preventive measures to avoid sexual transmission (condoms)? (Yes, No)

(e) Are your household contacts vaccinated against hepatitis B? (Yes, No)

Household contact interview

(a) General information

a.1 Age

a.2 Sex (Male, Women, Indeterminate)

a.3 Relationship with the patient

a.4 Place of birth (Spain, China, Other)

(b) Language barrier and Engagement to Local Health Programs

b.1 Attendance to Primary Care Physician (Yes, No)

b.2 Spanish Understanding (No, Limited, Yes)

b.3 Catalan Understanding (No, Limited, Yes)

(c) Knowledge about transmission mechanisms

c.1 Do you know the possibility of hepatitis B virus horizontal transmission through infected blood (sharing scissors, tooth brush, or manipulation of infected patients' wounds)? (Yes, No)

c.2 Do you know the possibility of hepatitis B virus transmission by sexual relationships? (Yes, No)

c.3 Do you know that hepatitis B virus can be transmitted from mother to child during pregnancy/delivery?(Yes, No)

(d) Use of preventive measures

d.1 Do you use of preventive measures to avoid exposure to infected blood (such as avoiding sharing personal items) (Yes, No)

d.2 Do you use preventive measures to avoid sexual transmission (condoms)? (Yes, No)

(e) Vaccination status (Vaccinated/ Not Vaccinated)

Supplementary table 2 : Multivariate analysis for knowledge on HBV transmission routes.

	**Knowledge on sexual transmission (OR 95% CI)**	**p**
Chinese	0,39 (0,12-1,34)	0.14
Male	5,02 (1,33-18,90)	**0.02**
	**Knowledge on vertical transmission (OR 95% CI)**	**p**
Chinese	4,93 (1,50-16,17)	**0.01**
Female	2,57 (0,91-7,22)	0.07
Age	0,97 (0,92-1,02)	0.19
Primary studies	1,39 (0,47-4,19)	0.55

Supplementary table 3. Knowledge and attitude regarding HBV transmission and comparison between Chinese vs Non- Chinese relative/household contacts.

**Relatives/household contacts**	**All N=398**	**Chinese N=240**	**Non Chinese N=158**	**p**
Spanish understanding				
No	37 (9%)	37 (15%)	0 (0%)	< 0.01
Limited	90 (23%)	69 (29%)	21 (13%)	
Good	271 (68%)	134 (56%)	137 (87%)	
Relationship to patient				< 0.01
Children	200 (50%)	138 (57%)	62 (39%)	
Partner	145 (36%)	81 (34%)	64 (41%)	
Others	53 (14%)	21 (9%)	32 (20%)	
Born in Spain		118 (49%)	152 (96%)	< 0.01
Knowledge on parenteral transmission n=268 (67%)				0.01
No	28 (10%)	24 (14%)	4 (4%)	
Yes	240 (90%)	147 (86%)	93 (96%)	
Knowledge on sexual transmission n= 262 (66%)				0.01
No	37 (14%)	32 (19%)	5 (5%)	
Yes	225 (86%)	133 (81%)	92 (95%)	
Knowledge on vertical transmission n=261 (66%)				0.25
No	45 (17%)	31 (19%)	14 (14%)	
Yes	216 (83%)	133 (81%)	83 (86%)	
Preventive measures (parenteral transmission) n=246 (62%)				0.02
No	66 (27%)	32 (21%)	34 (35%)	
Yes	180 (73%)	117 (79%)	63 (65%)	
Preventive measures (sexual transmission)** n=129 (32%)				0.01
No	44 (33%)	13 (19%)	31 (52%)	
Yes	85 (67%)	56 (81%)	29 (48%)	
Hepatitis B Vaccination				
No	74 (19%)	52 (22%)	22 (14%)	0.05

** only patient's partners were interviewed . For statistical analysis (X^2 test) individuals not willing to answer were not considered.

Supplementary Table 4 : Knowledge of transmission routes and preventive measures according to clinical and demographic characteristics in household contacts/relatives.

**Knowledge on mechanisms HBV transmission**										**Use of preventive measures for HBV transmission**					
**Variables N=398**	**Parenteral transmission**		**p**	**Sexual transmission**		**p**	**Vertical transmission**		**p**	**Parenteral transmission**		**p**	**Sexual transmission**		**p**
	Yes	No		Yes	No		Yes	No		Yes	No		Yes	No	
Age (Y)	45 (39-52)	49 (36-56)	0.37	46 (39-52)	46 (39-60)	0.33	45 (38-52)	47 (45-64)	**0.02**	43 (37-51)	46 (40-52)	0.25	41 (38-50)	46 (40-54)	0.05
Female<br>Male	138/154 (90%)<br>102/114 (89%)	16/154 (10%)<br>12/114 (11%)	0.97	130/152 (86%)<br>95/110 (86%)	22/152 (14%)<br>15/110 (14%)	0.85	124/150 (83%)<br>92/111 (83%)	26/150 (17%)<br>19/111 (17%)	0.96	106/146 (73%)<br>74/100 (74%)	40/146 (27%)<br>26/100 (26%)	0.80	49/76 (64%)<br>36/53 (68%)	27/76 (36%)<br>17/53 (32%)	0.68
Chinese<br>Non-Chinese	147/171(86%)<br>93/97 (96%)	24/171(14%)<br>4/97 (4%)	**0.01**	133/165 (81%)<br>92/97 (95%)	32/165 (19%)<br>5/97 (5%)	**0.01**	133/164 (81%)<br>83/97 (86%)	31/164 (19%)<br>14/97 (14%)	0.25	117/149 (79%)<br>63/97 (65%)	32/149 (21%)<br>34/97 (35%)	**0.02**	56/69 (81%)<br>29/60 (48%)	13/69 (19%)<br>31/60 (52%)	**0.01**

Supplementary Table 5: Influence of antiviral treatment and vaccination on preventive measures adopted by patients or contacts.

**Preventive measures in patients**	**Antiviral treatment**	**No treatment**	**p**
To avoid parenteral transmission (n=179)			0.13
No	9/74 (12%)	22/105(21%)	
Yes	65/74(88%)	83/105(79%)	
To avoid sexual transmission (n=181)			0.42
No	28/76(37%)	45/105(43%)	
Yes	48/76(63%)	60/105(57%)	
**Preventive measures in household contacts**	**Vaccinated**	**Non-vaccinated**	**p**
To avoid parenteral transmission (n=245)			0.26
No	46/194 (24%)	20/51 (39%)	
Yes	148/194 (76%)	31/51 (61%)	
To avoid sexual transmission (n=128)			0.48
No	30/90 (33%)	14/38 (37%)	
Yes	60/90 (67%)	24/38(63%)	
